# Response of Soil Respiration to Acid Rain in Forests of Different Maturity in Southern China

**DOI:** 10.1371/journal.pone.0062207

**Published:** 2013-04-23

**Authors:** Guohua Liang, Xingzhao Liu, Xiaomei Chen, Qingyan Qiu, Deqiang Zhang, Guowei Chu, Juxiu Liu, Shizhong Liu, Guoyi Zhou

**Affiliations:** 1 South China Botanical Garden, Chinese Academy of Sciences, Guangzhou, China; 2 University of Chinese Academy of Sciences, Beijing, China; DOE Pacific Northwest National Laboratory, United States of America

## Abstract

The response of soil respiration to acid rain in forests, especially in forests of different maturity, is poorly understood in southern China despite the fact that acid rain has become a serious environmental threat in this region in recent years. Here, we investigated this issue in three subtropical forests of different maturity [i.e. a young pine forest (PF), a transitional mixed conifer and broadleaf forest (MF) and an old-growth broadleaved forest (BF)] in southern China. Soil respiration was measured over two years under four simulated acid rain (SAR) treatments (CK, the local lake water, pH 4.5; T1, water pH 4.0; T2, water pH 3.5; and T3, water pH 3.0). Results indicated that SAR did not significantly affect soil respiration in the PF, whereas it significantly reduced soil respiration in the MF and the BF. The depressed effects on both forests occurred mostly in the warm-wet seasons and were correlated with a decrease in soil microbial activity and in fine root biomass caused by soil acidification under SAR. The sensitivity of the response of soil respiration to SAR showed an increasing trend with the progressive maturity of the three forests, which may result from their differences in acid buffering ability in soil and in litter layer. These results indicated that the depressed effect of acid rain on soil respiration in southern China may be more pronounced in the future in light of the projected change in forest maturity. However, due to the nature of this field study with chronosequence design and the related pseudoreplication for forest types, this inference should be read with caution. Further studies are needed to draw rigorous conclusions regarding the response differences among forests of different maturity using replicated forest types.

## Introduction

Acid rain, as a result of the dissolution of atmospheric sulfur dioxide (SO_2_) and nitrogen oxides (NO_x_) [1], [2] which originate mostly from anthropogenic activities such as industrial emission and automobile exhaust [3], [4], has been recognized as a worldwide environmental problem since the 1970s [5]. Studies have affirmed that acid rain can have detrimental effects on terrestrial and aquatic ecosystems [6]. As an important component of the terrestrial ecosystems, forests have also been deteriorating widely under the chronic stress of acid rain [5]. In some forests, for example, acid rain accelerated the leaching of nutrients from plant and soil [4], [7]–[10], stimulated Al^3+^ in soil solution which is toxic to the fine roots and the functioning of microbial community [11], and altered the forest species composition [12].

Although the emission of acidic gases in developed countries has declined in recent years, it is still increasing in many developing countries. Southern China was reported as the third largest acid rain area after Europe and the United States [13]. In this region, the annual average pH value of precipitation is generally below 4.5 in recent years, and precipitation with pH as low as 3.5 has been observed [14], [15]. Soil acidification due to acid rain has also occurred during the past 32–35 years, with a decrease of soil pH ranged from 0.1 to 1.0 pH units in some forests [16], [17]. One of the consequences of the fast soil acidification in this region is an alteration of the biogeochemical cycles and a challenge of the stability in the forest ecosystems [18].

Second only to gross photosynthesis (100–120 PgC yr^−1^), CO_2_ emission from soils (i.e. soil respiration, 68–100 PgC yr^−1^) is a major pathway in the global carbon cycle [19], [20]. Forest soil is an important source of CO_2_ in atmosphere [21], and soil respiration in forest is therefore a key process that underlies our understanding of the terrestrial carbon cycle [22]. Also, its response to environmental changes is an increasing concern [23]. Acid rain changes the conditions of soil and plant roots in forests, which is thought to have potential effects on soil respiration [24]. Although many efforts have been devoted to investigating the response of soil respiration in forests to acid rain, the results are often inconsistent. Decreases, increases, or unchanged in soil respiration after SAR treatments have been reported [25]–[27]. Moreover, up to now, most studies of SAR on soil respiration have been performed in temperate forest ecosystems in developed countries such as Europe and the United States [26]. There have been very few field studies on the response of soil respiration to acid rain in subtropical forests in southern China [28], [29]. Stand composition of forests in this region generally changes from coniferous to mixed coniferous and broad-leaved and to broadleaved in the process of forest succession [30] and therefore, forms forests of different maturity. Due to the different environmental conditions such as soil properties [31] and litter layer properties [32], we hypothesized that the response of soil respiration to acid rain would be different in forests of different maturity. For these reasons mentioned above, we carried out a field experiment to investigate the effects of SAR on soil respiration in three forests of different maturity at the Dinghushan Forest Ecosystem Research Station in southern China.

The aims of this study were to (1) determine if acid rain addition affects soil respiration in subtropical forests with high acidic soil, (2) compare the differences in the response of soil respiration to SAR among forests of different maturity, (3) and to identify possible mechanisms of the observed effects.

## Materials and Methods

### Ethics Statement

The study site is maintained by the South China Botanical Garden, Chinese Academy of Sciences. The location is within the Dinghushan Forest Ecosystem Research Station. All necessary permits were obtained for the described field study. The field study did not involve endangered or protected species. Data will be made available upon request.

### Site Description

The Dinghushan Forest Ecosystem Research Station, with an area of 1133 ha and an elevation ranging from 10 to 1000 m above sea level, is located in the middle part of Guangdong Province in southern China (112°30′–112°33′E, 23°09′–23°11′N). The area is characterized by a typical south subtropical monsoon climate with a distinct seasonal pattern. The annual mean temperature is 21°C with the maximum and minimum monthly mean temperature being 28.0°C in July and 12.6°C in January, respectively. The annual precipitation is 1927 mm, of which nearly 80% falls in the warm-wet season (April – September) and 20% in the cool-dry season (October – March) [33]. The annual average relative humidity is 82%. Acid rain was a threat in this area with an annual average pH value of precipitation proximately 4. 90 and a frequency of acid rain above 63% [34]. Bedrocks of the station are classified as Devonian sandstone and shale [35] and soils are classified as lateritic red earth (oxisol), loamy in texture, and acidic [36]. At the station, there are three types of forests of different maturity: the pine forest (PF), the mixed conifer and broadleaf forest (MF) and the broadleaf forest (BF) with age of more than 60, 110 and 400 years, respectively. They represent forests in young, transition, and old-growth stages in the region [30], [37]. During natural succession, heliophytes gradually invade the pine forests to form mixed forests, and mesophytes subsequently invade the mixed forests and eventually transform them into evergreen broadleaf forests [31]. The dominant species were *Pinus massoniana* in the PF, *Castanopsis chinensis*, *pinus massoniana* and *Schima superba* in the MF and *Cryptocarya concinna*, *Machilus chinensis* and *Cryptocarya chinensis* in the BF [32]. The main characteristics of the forests are listed in Table 1.

**Table 1:** Stand characteristics of the pine forest (PF), the mixed conifer and broadleaf forest (MF) and the broadleaved forest (BF) including the elevation, soil organic carbon (SOC), litterfall and accumulated litter.

Forest	PF	MF	BF
Elevation (m)	200–300	220–300	220–300
SOC** (g kg^−1^)	39.5±14.0	25.1±4.4	15.1±3.1
Litterfall* (g m^−2 ^yr^−1^)	598.3±56.1	701.2±83.8	893.2±59.2
Accumulated litter* (g m^−2^)	1058±121	686±96	575±77

* From Yan *et al.* (2006) [85].

** FromYi *et al.* (2007) [86]. The mean values of 0–15 cm soil layer.

### Experimental Treatments

Four SAR treatments were established by irrigating the plots with water of different pHs: CK (pH 4.5, the local lake water), T1 (water pH 4.0), T2 (water pH 3.5) and T3 (water pH 3.0). There were three replicates for each treatment. Twelve plots were established in each forest, with 10 m×10 m for each plot and surrounded by a 3 m wide buffer strip. All treatments were arranged randomly. According to Liu *et al.* [38], the pH value of precipitation in this region ranged from 4.36 during the dry period to 5.61 during the wet one. Considering the pH value of precipitation would probably decrease in the future, we set the T1 treatment as the lowest pH value observed in the natural rain and other two pH levels 0.5 unit lower each time. To reflect the real and the tendency of mole ratio of S:N according to the previous acid rain records, acidic solutions were prepared by adding a mixture of H_2_SO_4_ and HNO_3_ in a 1∶1 mole ratio to the local lake water. SAR treatments were initiated in June 2009 and were sprayed twice a month during the soil respiration measurement period. The simulated rainfall was applied to each plot below the canopy using a gasoline engine sprayer. The amount applied to each plot was 40 L per application. During the experimental period, the total H^+^ load each plot received was 9.6, 32, 96 mol ha^-l^ yr^-l^ in the T1, T2 and T3 treatments, respectively, which was equal to about 0.6, 2.0 and 6.0 times, respectively of that in the through-fall of the three forests.

### Field Sampling and Measurements

Ten months after the initial SAR application, soil respiration measurements were made twice a month from April 5, 2010 to March 20, 2012. Soil respiration was measured between 9∶00 am and 12∶00 am each time which were close to the daily mean, based on a study at an adjacent site where the diurnal pattern of soil respiration was measured [31]. Soil respiration was measured using a Li-8100 Infrared Gas Analyzer (Li-Cor Inc., Lincoln, NE, USA) with attached survey chamber. Two PVC collars (20 cm in diameter) were permanently anchored 5 cm into the soil in each SAR treatment plot. To eliminate the influence of plants on soil respiration, all living plants in the collars were removed before soil respiration measurement. Soil temperature (°C) at 5 cm depth and soil moisture (volumetric water content, %) of the top 5 cm soil layer were monitored simultaneously adjacent to each PVC collar using a digital thermometer and a PMKit [31], respectively.

To determine soil pH value, soil samples were collected in all forests in June and December of each year during the study period. We also collected soil samples in June 2011 for determining soil microbial biomass carbon. Two composite samples of four cores (2.5 cm inside diameter) from the upper soil layer (0–10 cm) were collected randomly from each plot. The composite samples were gently mixed and stored at 4°C until processing. Dead roots, litter and plant residues were picked out and the samples were passed through a 2-mm-mesh sieve. The soil pH values were measured using a glass electrode (1∶2.5 soil-water ratio) after shaking the samples to equilibration for approximately 30 min [39]. The soil microbial biomass carbon was estimated using DOC difference and a Kc factor of 0.33 [40], [41]. To determine fine root biomass, roots of 0–10 cm soil layer was collected by using a 6.8 cm diameter stainless-steel corer in June 2011. Two composite samples of three cores were randomly collected from each plot. The fine roots (diameter ≤2 mm) were separated by washing and sieving, dried at 60°C for 48 h and weighed [42].

### Statistical Analysis

Soil respiration in each pot was calculated as the mean of the two collar measurements, getting a sample size of three for each treatment in the analysis. Repeated measures ANOVA with Tukey’s HSD test was performed to examine the effects of SAR treatments on soil respiration, soil temperature, soil moisture and soil pH value among treatments for the study period. Standard t-test was used to test the seasonal (i.e. warm-wet vs. cool-dry seasons) difference in means of soil respiration rate, soil temperature and soil moisture. One-way ANOVA with Tukey’s HSD test was used to test the difference among treatments in means of annual soil respiration, soil pH value, soil microbial biomass carbon and fine root biomass, and among the control (CK) plots in three different forests. An exponential and a linear equation were used: *R* = *a*exp^(*bT* )^ (1); *R* = *cM*+*d* (2) [43], [44], where *R* is soil respiration rate (µmolCO_2_ m^−2^ s^−1^), *T* is soil temperature (°C), *M* is volumetric soil moisture (%) and *a*, *b, c and d* are constants fitted to the regression equation. The *Q*_10_ value which was defined as the difference in respiration rates over a 10°C interval was calculated using the exponential relationship between soil respiration and soil temperature (*Q*_10_ = exp^(10*b*)^) [45], [46]. One-way ANOVA test was also used to compare the regression slopes (*b* values) among treatments. Data analyses were carried out using the SAS software (SAS Version 8.0, SAS Institute Inc. Cary, NC, USA). Statistical significant differences were set with *p*<0.05 unless otherwise stated.

## Results

### Soil Temperature and Soil Moisture

**Figure 1:**
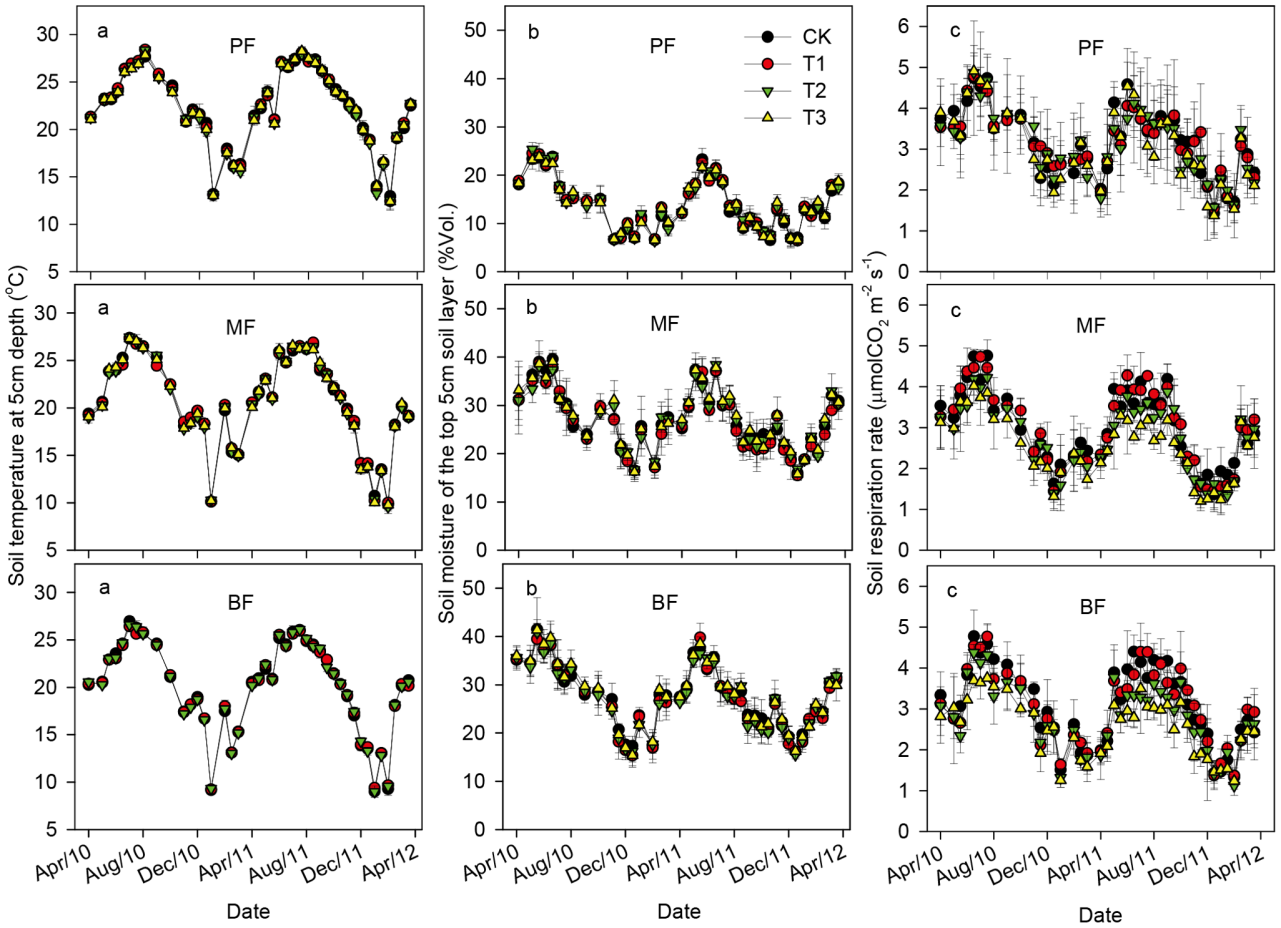
Seasonal dynamics of soil temperature, soil moisture and soil respiration under different SAR treatments in the pine forest (PF), the mixed conifer and broadleaf forest (MF) and the broadleaved forest (BF). (a) soil temperature at 5 cm depth; (b) volumetric soil moisture of the top 5 cm soil layer; (c) soil respiration rate. Error bars are standard errors of the mean (n = 3 for all the treatments). The treatments are: CK = control, T1 = pH 4.0, T2 = pH 3.5, T3 = pH 3.0.

Soil temperature and soil moisture (Fig. 1 a, b) exhibited strong seasonal patterns for all treatments in the three forests. Soil was warmer and wetter from April to September (the warm-wet season) than that from October to March of the next year (the cool-dry season) (*p*<0.05 for all). Annual mean soil temperature in the control (CK) plots was in the order of PF (22.30±0.13°C)>MF (20.67±0.06°C)>BF (20.00±0.04°C) (*p*<0.05). Annual mean soil moisture in the control (CK) plots was not significantly different between the MF (26.98±0.61%) and the BF (27.68±0.71%), while the PF (13.76±0.24%) was significantly lower than the MF and the BF (*p*<0.05). There was no treatment effect on neither soil temperature nor soil moisture (*p*>0.05 for both in all forests) during the study period.

### Soil Respiration

Soil respiration in different treatments also followed a clear seasonal pattern in all three forests during the study period, with significantly higher rates in the warm-wet seasons and lower rates in the cool-dry ones (*p*<0.05) (Table 2; Fig. 1 c). Mean annual soil respiration in the control (CK) plots was 4.30±0.35, 4.20±0.20 and 4.28±0.13 kgCO_2_ m^−2^ yr^−1^ in the PF, the MF and the BF, respectively, and there was no significant difference among three forests (*p*>0.05) (Table 3; Fig. 1 c).

**Table 2:** Mean soil respiration rate in the pine forest (PF), the mixed conifer and broadleaf forest (MF) and the broadleaved forest (BF) under different SAR treatments (mean ± standard deviations).

Forest	Season	CK	T1	T2	T3
PF	Wet season	3.69±0.21 a*	3.63±0.59 a*	3.60±0.55 a*	3.61±0.06 a*
	Dry season	2.46±0.23 a*	2.56±0.67 a*	2.44±0.41 a*	2.27±0.25 a*
MF	Wet season	3.72±0.16 a*	3.78±0.11 a*	3.41±0.03 b*	3.12±0.20 b*
	Dry season	2.27±0.18 a*	2.24±0.15 a*	2.12±0.28 a*	1.95±0.13 a*
BF	Wet season	3.72±0.16 a*	3.64±0.28 a*	3.29±0.32 ab*	3.03±0.05 b*
	Dry season	2.43±0.03 a*	2.49±0.06 a*	2.27±0.27 a*	2.09±0.12 a*

n = 3 for all the treatments. Mean values within a row with different lowercase letter have significant treatment differences at *p = *0.05 level. Means values within each column indicated by the asterisk (*) show significant seasonal differences at *p* = 0.05 level. The treatments are: CK = control, T1 = pH 4.0, T2 = pH 3.5, T3 = pH 3.0. Unit: µmolCO2 m^−2^ s^−1^.

**Table 3:** Annual soil respiration (R) and its % decreased for each year in the pine forest (PF), the mixed conifer and broadleaf forest (MF) and the broadleaved forest (BF) under different SAR treatments (mean ± standard deviations).

		CK	T1		T2		T3	
Forest	Year	R	R	% decreased	R	% decreased	R	% decreased
PF	The 1^st^ year	4.49±0.59 a	4.57±0.37 a	−1.73	4.54±0.42 a	−1.20	4.44±0.44 a	1.12
	The 2^nd^ year	4.11±0.11 a	4.01±1.09 a	2.55	3.91±0.09 a	4.95	3.82±0.67 a	7.21
	Mean	4.30±0.35 a	4.29±0.72 a	0.32	4.23±0.26 a	1.74	4.13±0.55 a	4.03
MF	The 1^st^ year	4.41±0.07 a	4.35±0.05 a	1.37	4.04±0.35 a	8.28	3.84±0.49 a	12.86
	The 2^nd^ year	3.99±0.36 a	4.06±0.20 a	−1.65	3.70±0.11 ab	7.34	3.30±0.17 b	17.23
	Mean	4.20±0.20 a	4.20±0.12 a	−0.07	3.87±0.21 ab	7.83	3.61±0.24 b	13.99
BF	The 1^st^ year	4.40±0.21 a	4.33±0.32 a	1.56	4.07±0.27 a	7.60	3.83±0.18 a	12.99
	The 2^nd^ year	4.16±0.21 a	4.23±0.16 a	−1.60	3.73±0.52 ab	10.32	3.38±0.14 b	18.79
	Mean	4.28±0.13 a	4.28±0.24 a	0.01	3.90±0.37 ab	8.92	3.61±0.09 b	15.81

n = 3 for all the treatments. Mean values within a row with different lowercase letter have significant treatment differences at *p = *0.05 level. The treatments are: CK = control, T1 = pH 4.0, T2 = pH 3.5, T3 = pH 3.0. % decreased = 100 ((R of the CK treatment – R of each treatment)/R of the CK treatment) % (in the same time period); negative values within the column means % increased. The 1^st^ year: April 2010 to March 2011; The 2^nd^ year: April 2011 to March 2012; Mean: April 2010 to March 2012. Unit: kgCO2 m^−2^ yr^−1^ for R.

The sensitivity of the response of soil respiration to SAR showed an increasing trend with the progressive maturity of three forests. Compared with the CK treatment, mean annual soil respiration was 0.3, 1.7 and 4.0% lower in the T1, T2 and T3 treatments, respectively in the PF. The repeated measures ANOVA showed that SAR did not affect soil respiration in the PF (*p = *0.97), but it significantly reduced soil respiration in the MF and the BF(*p = *0.02 and 0.01, respectively). Compared with the CK treatment, mean annual soil respiration in the T1, T2 and T3 treatments was changed by −0.1, 7.8 and 14.0%, respectively in the MF and 0, 8.9 and 15.8%, respectively in the BF (Table 3; Fig. 1 c). Both in the MF and the BF, there were no significant differences among the CK, T1 and T2 treatments, while the T3 treatment was significantly lower than the CK and T1 treatments (*p*<0.05 for both). These negative effects were evident in the warm-wet seasons ((*p*<0.01 and *p = *0.02 in the MF and the BF, respectively), but not in the cool-dry ones (*p*>0.05 for both) (see Table 2; Fig. 1 c).

In addition, by analyzing the annual soil respiration of each year, we found that these negative effects had been strengthened over time. Compared with the CK treatment, there were −1.73–1.12, 1.37–12.86 and 1.56–12.99% lower in the acid treatment plots in the PF, the MF and the BF, respectively (*p*>0.05 for all) in the first year, and 2.55–7.21, 1.65–17.23 and 1.60–18.79% lower in the PF, the MF and the BF, respectively (significantly different in the MF and the BF with *p*<0.05 for both) in the second year (Table 3; Fig. 1 c).

**Figure 2:**
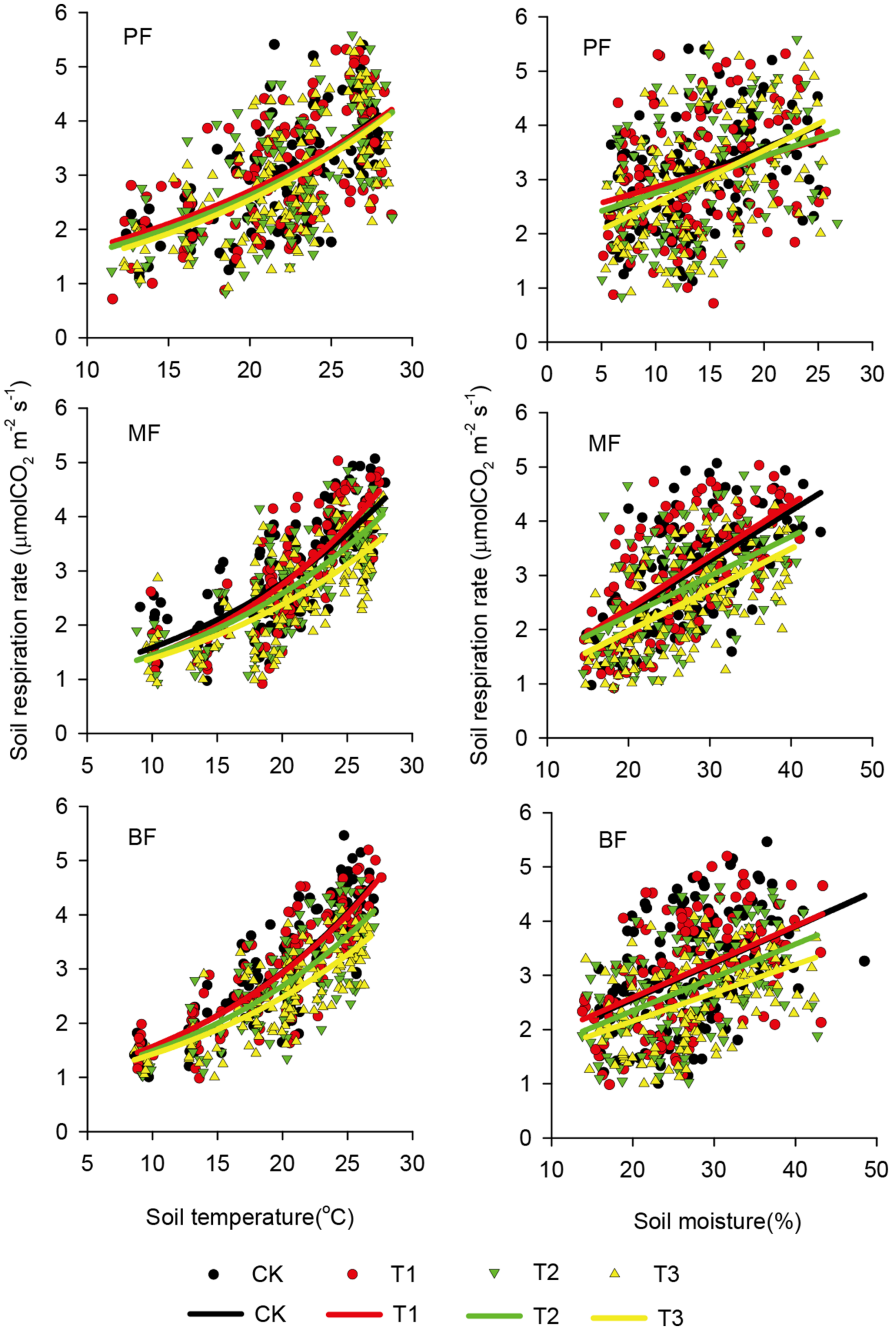
Relationship of soil respiration rate with soil temperature or soil moisture under different SAR treatments in the pine forest (PF), the mixed conifer and broadleaf forest (MF) and the broadleaved forest (BF). n = 126 for all the treatments. The treatments are: CK = control, T1 = pH 4.0, T2 = pH 3.5, T3 = pH 3.0.

In all treatments of the three forests, soil respiration exhibited significantly positive exponential relationships with soil temperature (*p*<0.01, with *R* ^2^ ranging from 0.34 to 0.41, 0.51 to 0.67 and 0.59 to 0.71 in the PF, the MF and the BF, respectively) and significantly positive linear relationships with soil moisture (*p*<0.01, with *R* ^2^ ranging from 0.10 to 0.23, 0.25 to 0.45 and 0.19 to 0.22 in the PF, the MF and the BF, respectively) (Table 4; Fig. 2). The mean temperature sensitivity (*Q*_10_) values for the CK, T1, T2 and T3 treatments were 1.75, 1.75, 1.69 and 1.71 in the PF, 1.80, 1.88, 1.76 and 1.72 in the MF and 1.88, 1.87, 1.80 and 1.74 in the BF, respectively (Table 4). *Q*_10_ value showed a declining trend with the intensification of acidification, although it was not statistically significant.

**Table 4:** Model for relationships between the soil respiration (*R*) and soil temperature at 5 cm depth (*T*) and volumetric soil moisture of the top 5 cm soil layer (*M*) in the pine forest (PF), the mixed conifer and broadleaf forest (MF) and the broadleaved forest (BF).

Model		*R* = *a*exp^(*bT*)^ *				*R = aM*+*b* *		
Forest	Treatment	*a*	*b*	*Q*_10_	*R^2^*	*a*	*B*	*R^2^*
PF	CK	0.8375±0.3837	0.0585±0.0177 a	1.75	0.41	0.0698±0.0125	2.0850±0.1520	0.18
	T1	1.0012±0.6896	0.0588±0.0455 a	1.75	0.34	0.0577±0.0431	2.1087±0.6338	0.10
	T2	0.7604±0.1062	0.0569±0.0121 a	1.69	0.35	0.1071±0.0369	1.3543±0.7017	0.12
	T3	0.9266±0.3183	0.0518±0.0066 a	1.71	0.36	0.0504±0.0284	2.2875±0.8416	0.23
MF	CK	0.7807±0.2344	0.0595±0.0127 a	1.80	0.57	0.0873±0.0228	0.5439±0.8123	0.40
	T1	0.7462±0.1461	0.0646±0.0069 a	1.88	0.67	0.0918±0.0110	0.5101±0.3868	0.39
	T2	0.9138±0.2138	0.0557±0.0122 a	1.76	0.61	0.0770±0.0054	0.5489±0.1247	0.25
	T3	0.8523±0.1124	0.0490±0.0085 a	1.72	0.51	0.0666±0.0193	0.5011±0.4646	0.35
BF	CK	0.7340±0.1494	0.0687±0.0111 a	1.88	0.70	0.0898±0.0112	0.6235±0.4818	0.19
	T1	0.8353±0.1566	0.0630±0.0082 a	1.87	0.71	0.0870±0.0096	0.7855±0.2888	0.22
	T2	0.8724±0.1447	0.0546±0.0117 a	1.80	0.59	0.0805±0.0446	0.5894±0.8082	0.21
	T3	0.8069±0.1378	0.0539±0.0065 a	1.74	0.64	0.0632±0.0274	0.7312±0.9059	0.22

* *p*<0.01 in all treatments of all forests.

n = 126 for all the treatments. *R^2^* is the determination of coefficient. The treatments are: CK = control, T1 = pH 4.0, T2 = pH 3.5, T3 = pH 3.0.

### Soil pH Value

**Figure 3:**
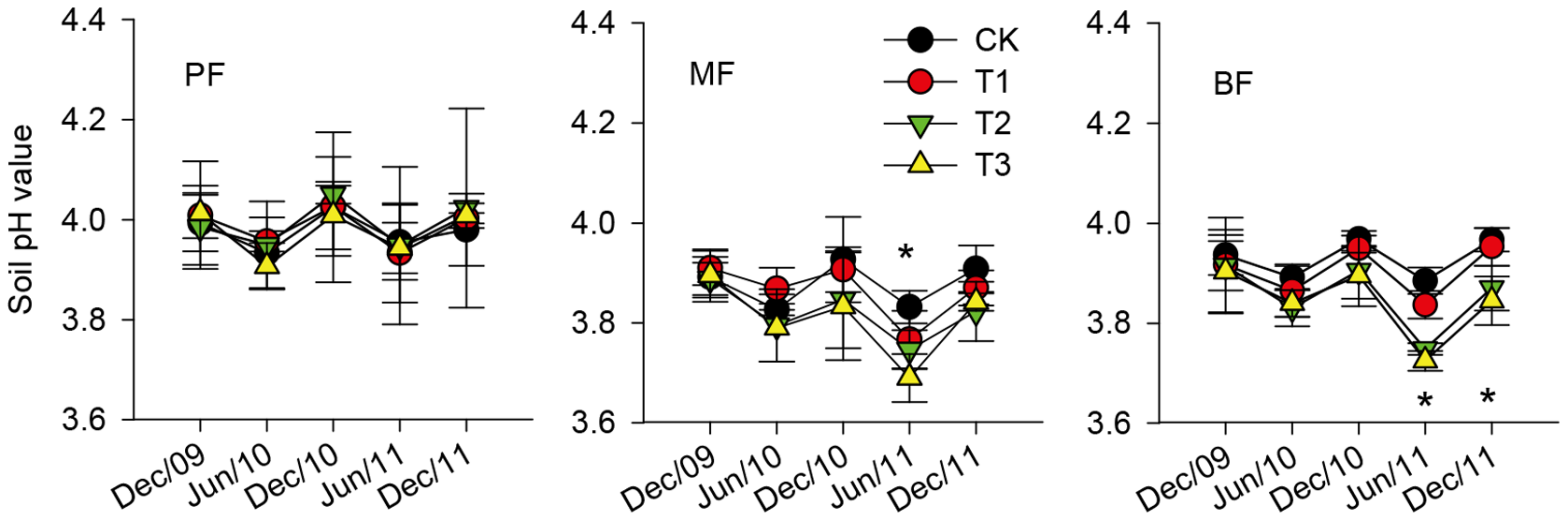
Dynamics of soil pH value under different SAR treatments in the pine forest (PF), the mixed conifer and broadleaf forest (MF) and the broadleaved forest (BF). Error bars are standard errors of the mean (n = 3 for all the treatments). The asterisk (*) indicates significant difference among treatments at *p = *0.05. The treatments are: CK = control, T1 = pH 4.0, T2 = pH 3.5, T3 = pH 3.0.

From December 2009 to December 2011, mean soil pH values in the control (CK) plots were 3.97±0.06, 3.88±0.01 and 3.90±0.01 in the PF, the MF and the BF, respectively (Fig. 3). Mean soil pH values in the MF and the BF were significantly lower than that in the PF (*p*<0.05). The repeated measures ANOVA showed that SAR did not affect soil pH value in the PF (*p = *0.997), but it significantly reduced soil pH value in the BF (*p = *0.01) and the reduction was marginally significant in the MF (*p = *0.07). Also, we observed that these declining trends in the BF and the MF had been strengthened over time. The significant differences of soil pH value among treatments were found in the BF in June 2011 and December 2011(the pH values in the T3 and T2 treatments were significant lower than those in the CK and T1 treatments in both time with *p*<0.05) and in the MF in June 2011(the pH value in the T3 treatment was significant lower that in the CK treatment with *p*<0.05).

### Soil Microbial Biomass Carbon and Fine Root Biomass

**Figure 4:**
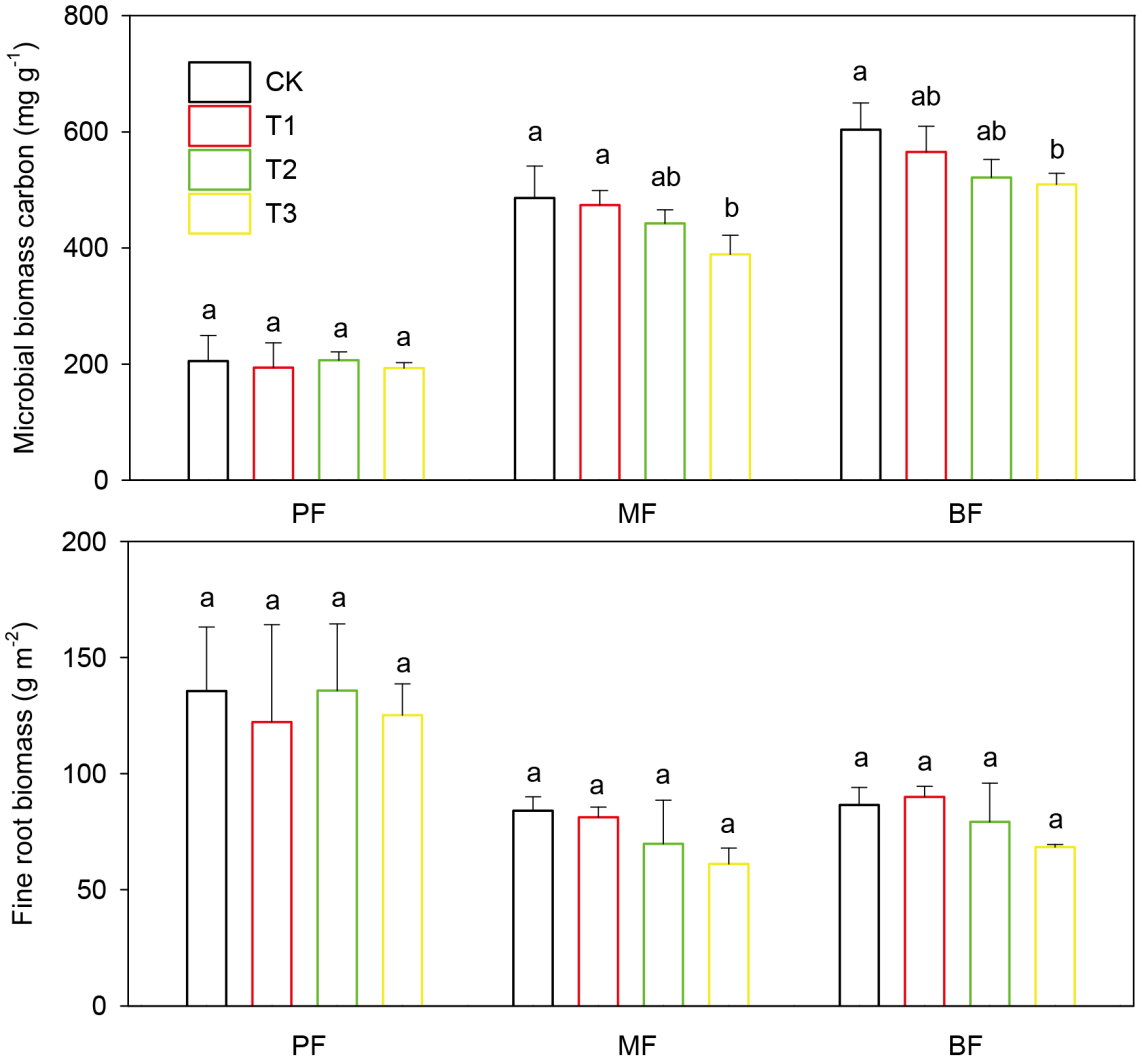
Soil microbial biomass carbon and fine root biomass under different SAR treatments in the pine forest (PF), the mixed conifer and broadleaf forest (MF) and the broadleaved forest (BF). Error bars are standard errors of the mean (n = 3 for all the treatments). Different lowercase letters denote significant difference (*p = *0.05) between treatments. The treatments are: CK = control, T1 = pH 4.0, T2 = pH 3.5, T3 = pH 3.0.

Mean microbial biomass carbon in the control (CK) plots in the MF (486.12±55.03 mg kg^−1^) and the BF (603.76±46.18 mg kg^−1^) was significantly higher than that in the PF (205.42±44.00 mg kg^−1^) (*p*<0.05). On the contrary, fine root biomass in the control (CK) plots in the MF (84.03±6.00 g m^−2^) and the BF (86.53±7.54 g m^−2^) was significantly lower than that in the PF (135.53±27.60 g m^−2^) (*p*<0.05) (Fig. 4). Compared with the CK treatment, the microbial biomass carbon was 2.5, 9.0 and 20.0% lower in the T1, T2 and T3 treatments, respectively in the MF and 6.4, 13.6 and 15.6% lower, respectively in the BF, and significant differences were found among the T3, T1 and CK treatments in the MF and between the T3 and CK treatments in the BF (*p*<0.05 for both). Although the fine root biomass had the same decreasing tendency with 3.3, 16.9 and 27.3% lower in the T1, T2 and T3 treatments, respectively compared with the CK treatment in the MF and 4.0, 8.5 and 21.0% lower, respectively in the BF, the differences among treatments were not statistically significant in both forests (*p*>0.05 for both). However, there were no significant differences among treatments in the microbial biomass carbon and the fine root biomass in the PF (*p*>0.05 for all).

## Discussion

### Soil Respiration of the Control (CK) Plots in Three Forests

The mean annual soil respiration in the control (CK) plots of our forests (Table 3) was in the same range as a previous study in adjacent forests [31] and as some similar studies worldwide [47]–[49]. The mean temperature sensitivity (*Q*_10_) values for soil respiration in the control (CK) plots (Table 4) was similar to the reported values in a subtropical forest in China (1.75–2.55, [50]), but lower than those in a temperate forest (2.6–3.2, [51]). Soil respiration in the control (CK) plots was not significantly different among the three forests of different maturity, which may be related to the compensation mechanism discussed below. Soil respiration mainly consists of two components: autotrophic (root) respiration and heterotrophic (microbial) respiration, which are related to the amount of living root biomass and the decomposition of litter and soil organic matter, respectively [52], [53]. Although the microbial biomass and SOC in the control (CK) plots in the PF were lower than those in the MF and the BF, the fine root biomass was higher in the PF (Table 1; Fig. 4). Therefore, the relatively lower heterotrophic respiration could be compensated by the higher autotrophic respiration in the PF.

### Effect of Soil Temperature and Soil Moisture on the Seasonality of Soil Respiration

Soil respiration in all treatments of the three forests exhibited strong seasonal patterns with higher rates in the warm-wet seasons and lower rates in the cool-dry ones (Table 2; Fig. 1 c), which is consistent with the results reported in some subtropical forests [31], [54]–[56] and in some temperate forests [21], [57], [58]. In addition, similar to the related studies in some warm and wet forests [42], [49], positive exponential relationships between soil respiration and soil temperature, as well as positive linear relationships between soil respiration and soil moisture have been found in our forests (Table 4; Fig. 2). The dual temperature and moisture controls on soil respiration in this study may be related to the monsoon tropical climate of our studied region with high temperature occurs simultaneously with high moisture [37]. Therefore, high plant growth and soil microbial activity in the warm-wet season can stimulate greater soil respiration in this region [59].

### Effects and Mechanisms of SAR on Soil Respiration

Our results demonstrated that soil respiration in all three forests were depressed after exposure to SAR. This result was consistent with several laboratory and field experimental results [29], [58], [60], indicating that the response of soil respiration was susceptible to acid rain in subtropical forests. However, the sensitivity of the response of soil respiration to SAR was different among the three forests. The response of soil respiration to SAR was less significant in the PF than those in the MF and the BF (4.0, 14.0 and 15.8% lower in the T3 treatment compared with the CK treatment, respectively) and the differences among treatments were significant in the MF and the BF (Table 3; Fig. 1 c).

Several mechanisms may help explain the depression of soil respiration after SAR in the MF and the BF and that mostly occurred in the warm-wet season. First, soil acidification under SAR may decrease heterotrophic respiration from the microbial community. After SAR treatment for 25 months, mean soil pH values in the T3 treatment in both forests were significantly lower than those in the CK treatment (Fig. 3), which indicated the aggravation of soil acidification. Many related studies in temperate and subtropical forests also suggest that SAR decreases soil pH value [29], [61], [62]. Lower soil pH value due to the toxicity of high H^+^ loads can change the population, community structure and biological activity of soil decomposers [63]. In this study, we found that soil microbial biomass carbon in the warm-wet season significantly decreased in the T3 treatment both in the MF and the BF (Fig. 4), which was consistent with several studies in temperate and subtropical forests [64]–[66]. This suggests that microbial activity was restrained under the stress of acid rain. The reduction of microbial activity, on one hand, would slow the mineralization and decomposition rates of soil organic matter, thus inhibiting CO_2_ emission from soil [62]; on the other hand, litter decomposition would be inhibited, which therefore, leads to the reduction of microbial CO_2_ production from litter [67]. Many studies have also reported that the litter decomposition rates in subtropical and temperate area slowed down under the stress of SAR [68], [69].

Second, autotrophic respiration from plant roots may decrease after soil acidification. It was reported that fine root biomass was significantly correlated with soil respiration rate [22], and SAR decreased fine root biomass and inhibited seedling growth [70]. In one way, cumulative effects of acid rain will lead to nutrient leaching [71]–[73] as the amount of H^+^ cation in the soil increases and they replace the basic cation in the argilo-humic complex. A nutrient depletion will then follow in the long term and the growth of plants can be affected [38]. In another way, when the soil pH value decreases, the concentration of free moving metallic ions (e.g. Al^3+^, Mn^2+^) will increase [74], [75]. Those metals show very high degrees of toxicity for many plants [76], [77], and thus affect root respiration [14]. In this study, the fine root biomass in the warm-wet season in the MF and the BF revealed a negative response to the increasing level of SAR (Fig. 4), which was also accompanied by the decreased CO_2_ loss from the soils.

Under the stress of acid rain, the soil will be acidified gradually [34], [75]. The declining trends of soil pH value in the MF and the BF had been strengthened over time during the study period (Fig. 3), which also indicated that the soil acidification under SAR was a gradual process in these two forests. Therefore, the negative effects of SAR on soil respiration had been strengthened over time accordingly (Table 3). Furthermore, we found an effect of SAR on the temperature response of soil respiration, with a decline in *Q*_10_ in all forests (Table 4). This result suggests that SAR would decrease the temperature sensitivity of respiration. As a respiratory substrate, root biomass plays an important role in the response of soil respiration to soil temperature; temperature sensitivity of soil respiration decreases when the substrate supply is low [59]. Therefore, the reduction of root biomass in this study could result in lower temperature sensitivity. Although *b* values for the exponential curve among treatments were not significantly different in all forests, we suggest that this decrease would be more significant under the continued SAR in the future. Moreover, the decline of the temperature sensitivity of respiration was increasingly pronounced with the progressive maturity of three forests, which indirectly indicated the same trend in the sensibility of the response of soil respiration to SAR.

### Mechanisms of the Different Responses of Soil Respiration among Three Forests

We suspect that the different responses of soil respiration to SAR among three forests may result from their differences in soil and in litter layer which, ultimately, are caused by the differences in maturity. First, acid buffering abilities of soil are different among the three forests. The decrease of soil pH is the most direct indicator of soil acidification [62]. At our study sites, although the soil pH values of 0–10 cm depth were highly acidic with mean pH values below 4 in the control (CK) plots of all forests, soil acidification was aggravated when exposed to an addition of H^+^. This may relate to the low base saturation (BS, less than 10%, [78]) in the soil of our forests, as the BS can reflect the buffering capacity of soil to acid rain [79]. However, unlike the situations in the MF and the BF, there was no significant difference among treatments in the soil pH value in the PF (Fig. 3). Yu *et al.* [80] pointed out that soil acidification under acid rain is closely related to the degree of pH value of original soil, that is the higher the original soil pH, the smaller the affect of soil acidification by acid rain. Soil pH value in the control (CK) plots in the PF was significantly higher than those in the MF and the BF (Fig. 3), indicating that the soil in the PF was less susceptible to acid rain. In addition, the BS in soil was decrease with the progressive maturity of three forests [78], which also suggested that the acid buffering ability of soil was the highest in the PF.

Second, acid buffering abilities of litter layer are different among the three forests. The litter layer is not only a source of soil respiration but also an influence of soil respiration by indirect effects on biological processes in the underlying soil [32]. The decaying litter above the surface of soil can mitigate the effects of acid rain by the exchange between the base cation in the litter and H^+^ in the acid rain [81]. Although the annual litterfall decreased with the progressive maturity of three forests, the amount of litter present was in the opposite trend (PF>MF>BF) (Table 1) due to the decreased trend of decomposition rate [82]. Therefore, the acid buffering ability of litter layer was more evident in the PF than those in the MF and the BF. The result of Liu *et al.* [83] also suggested that the acid buffering ability of litter layer was best in the pine forest among six forest types in southwestern Subtropical region of China. The acid buffering ability of soil and litter layer in the PF mitigated the acidification of soil and the reductions of soil microbial activity and fine root biomass under SAR, thus alleviated the depression of soil respiration.

### Limitation of the Study

In this study, we selected three typical forest types of different maturity in southern China, tested the effects of acid rain on soil respiration, and compared the response differences among forests of different maturity. One shortcoming of this study was that we did not have true replication for forest age; this is a common feature of many chronosequence designs [84] but means we cannot draw formal inferences about the effect of age on the interaction between soil respiration and SAR. Thus, the inferences regarding the response differences among forest types should be read with caution. Further studies are needed to draw rigorous conclusions regarding the response differences among forests of different maturity using replicated forest types.

### Conclusions

SAR did not affected soil respiration in the PF during the study period, but significantly reduced soil respiration in the MF and the BF. These depressed effects on both forests occurred mostly in the warm-wet seasons and were correlated with the decreases in soil microbial biomass carbon and fine root biomass caused by soil acidification under SAR. The sensitivity of the response of soil respiration to SAR showed an increasing trend with the progressive maturity of three forests. This result indicated that the depressed effect of acid rain on soil respiration in southern China may be more pronounced in the future, as the young forests that are currently dominant in the region due to the widespread afforestation in recent years would gradually become mature. Further studies are still needed to draw rigorous conclusions regarding the response differences among forests of different maturity using replicated forest types.
